# Quantification of whisky congeners by ^1^H NMR spectroscopy

**DOI:** 10.1002/ansa.202000063

**Published:** 2020-07-27

**Authors:** Marc Stockwell, Ian Goodall, Dušan Uhrín

**Affiliations:** ^1^ EaStCHEM School of Chemistry Joseph Black Building University of Edinburgh Edinburgh UK; ^2^ The Robertson Trust Building Research Avenue North, Riccarton The Scotch Whisky Research Institute Edinburgh UK

**Keywords:** qNMR, quantification of congeners, quantitative NMR, Scotch Whisky

## Abstract

Whisky is a complex mixture made up of thousands of compounds originating in different stages of its production. Analysis of whisky congeners is critical to our understanding of the manufacturing process, quality control, and the detection of counterfeit products. The current chromatographic methods have a long analysis time, can require milliliters of sample and may not detect all required compounds in a single analysis. We have demonstrated that the majority of the whisky congeners of interest can be analyzed using ^1^H NMR spectroscopy in a single session using 500 μL of sample with the addition of 100 μL of buffer. We addressed two issues with this application of NMR: sensitivity and complexity of spectra. The sensitivity issues were solved by using a highly sensitive 600 MHz instrument equipped with a cryoprobe. To achieve consistent quantitative analysis of overlapping signals, Chenomx software was used. This allowed successful determination of the absolute concentration of 13 of the 21 studied whisky congeners with an average relative difference from nominal concentration of 6.4% and a standard deviation of 5.0%. Some compounds such as iso‐amyl acetate and *n*‐butanol were not accurately quantifiable due to their low concentration and overlapping peaks with those of more concentrated compounds. Scopoletin, lactose, sucrose, and maltose were not detectable in whisky samples, but they were accurately quantified in model mixtures. At higher concentrations, these compounds could be accurately quantified in whisky samples. Overlap of glucose and fructose signals led to >10% deviations from nominal concentration values. The limits of quantification (LOQ) and limits of detection (LOD) for each analyte were determined, with the LOD varying between 10 and 20 μM for the major volatile congeners, 1 to 5 μM for maturation related congeners, and 10 to 30 μM for carbohydrates.

## 1 INTRODUCTION

Whisky (or whiskey) is produced all over the world, from Japan to Ireland, but Scotch Whisky demonstrates global dominance with 1.3 billion bottles being shipped from Scotland to 180 markets worldwide each year, creating export revenues of £4.9 billion as of 2019.^1^ To protect this multi‐billion pound industry, the production of Scotch Whisky is regulated by strict guidelines laid down in the 2009 Scotch Whisky Regulations. The regulations stipulate among other things the allowed ingredients (cereals, water, yeast, and plain caramel coloring), the fact that Scotch Whisky must be matured in oak casks in Scotland for no less than 3 years, and bottled at a minimum strength of 40% alcohol by volume (ABV).^2^

Whisky production starts with three simple ingredients: cereals, water, and yeast. These go through a series of production steps, starting with mashing. This is where the starch in the cereals is hydrolyzed to create a fermentable mixture of carbohydrates (the wort).^3^ Malt Scotch Whisky is made from 100% malted barley; grain Scotch Whisky is made from malted barley and other cereals, typically wheat or maize. Fermentation follows by adding yeast that converts the carbohydrates to ethanol and carbon dioxide.^4^ The alcoholic solution produced, approximately 8‐10% ABV is then distilled. Malt Scotch Whisky goes through a two (or three) stage batch distillation process producing a new make spirit at about 70% ABV;^5^ Grain Scotch Whisky is typically distilled to a much higher strength using continuous column distillation.^6^ The resulting distillates are stored in oak casks in Scotland for a desired period (a minimum of 3 years) to mature,^7^ before being blended and bottled as Scotch Whisky.^8^

The blending process is an important part of the production of a vast array of different whisky brands. These can be classified into a handful of categories: Single Malt Scotch Whisky, which is a malt Scotch Whisky from a single distillery; Single Grain Scotch Whisky, which is a grain Scotch Whisky from a single distillery; Blended Malt or Blended Grain that are, respectively, a blend of two or more malt or grain Scotch Whiskies distilled at different distilleries; and, by volume, the most popular type of Scotch Whisky, the Blended Scotch Whisky, which is a blend of one or more Single Malt Scotch Whiskies with one or more Single Grain Scotch Whiskies.

During fermentation, distillation, and maturation, various compounds, collectively known as congeners, are embedded in the ethanol/water matrix, constituting a complex mixture. The ability to quantify the amount of a known analyte as part of a mixture is very important for many industries, especially when the product is destined for human consumption, for example, as food or a beverage. This is required in order to introduce quality control to a product or a procedure and at the same time monitor undesired compounds, for example, those that may have potential negative flavor profiles.^9^ Currently, analysis of congeners in the whisky industry and whisky research makes use of chromatographic methods, such as gas chromatography (GC) and liquid chromatography (LC), often coupled with a spectroscopic or spectrometric detector, such as UV‐Vis (UV) or mass spectrometry (MS).^10^, ^11^, ^12^ These methods are used due to their versatility, affordability of instrumentation, and ease of use. However, they have several drawbacks. Development of standard operating protocols can be long and time consuming, requiring milliliters of sample and extensive preparation of standards. The main drawback of chromatographic methods, however, is that they rely on unique retention times of the analytes for their identification. This can lead to misclassification, or misquantification if the peak being measured is not purely the compound of interest. Spectroscopic methods, such as UV‐Vis spectroscopy,^13^ infrared spectroscopy,^14^ or Raman spectroscopy,^15^ tend to focus on the search for adulterants by identifying deviations from overall trends expected in whisky spectra; their usage in analysis of congeners is limited.

NMR reports in this area are rather sparse. Authors mainly used chemometric approaches and nontargeted analysis to establish authenticity,^16^ detect counterfeit products,^17^, ^18^ model whisky production parameters, and to identify whisky congeners.^18^ A parahydrogen enhanced NMR chemosensing approach was proposed for the quantitative determination of specific flavor components in cask strength single‐malt Scotch Whisky.^19^ Applied to other alcoholic beverages, Fotakis and Zervou^20^ used a chemometric approach to classify Greek grape marc spirits. A targeted analysis of alcoholic drinks by NMR has been reported for the detection of ethyl carbamate in spirits.^21^

There are two main challenges facing profiling whisky by ^1^H NMR. The first ‐ the signals of the two main compounds in whisky, water and ethanol, dominate the spectra and need to be suppressed using a form of “solvent suppression.” A method developed by Kew et al^22^ for the use on cryoprobe instruments and suppression of the ^13^C satellite signals of ethanol, which can also dwarf the signals of interest, was used throughout this work. The second challenge for the quantification of whisky congeners by NMR is an overlap of multiple signals of individual compounds in a mixture as complex as whisky. This makes identification and particularly quantification of compounds non‐trivial, as exemplified by the failure to quantify the ethyl carbamate in a previous study.^21^

The objective of this study thus was to investigate if routinely analyzed Scotch Whisky congeners can be quantified during a short NMR session using a small sample volumes (500‐600 μL) and minimum pretreatment in combination with targeted quantitative profiling provided by Chenomx NMR Suite 8.4.

## 2 MATERIALS AND METHODS

### 2.1 Standards and samples

Pure compound standards (major volatile and maturation related congeners sourced from, Greyhound Chemicals, Merseyside, UK and Sigma‐Aldrich, Missouri, USA, respectively) were supplied by the Scotch Whisky Research Institute (SWRI) in 40% ABV ethanol:water solutions at concentrations of 0.5 mg/mL for the maturation related congeners and 50 mg/mL for the major volatile congeners. A stock solution (0.8‐1.6 mg/mL) of each carbohydrate (Sigma‐Aldrich, Missouri, USA) was prepared by dissolving the pure compound in a 40% ABV ethanol:water solution. Prior to use, these standards of known concentrations were diluted to the desired concentration using a 40% ABV ethanol:water solution. The water and ethanol (≥99.8% purity, with a 16 μM methanol contaminant) used in the preparation of all standards and model mixtures were sourced from Fisher Scientific, Pennsylvania, USA. SWRI also provided 49 Scotch Whisky samples from their 2018 standard sample set, curated to broadly represent the Scotch Whiskies produced that year. A further 37 samples of cask and bottle strength whiskies were also supplied, extending the sample set to 86. The samples were received with a set of results obtained by SWRI using internal operating procedures accredited by UKAS (United Kingdom Accreditation Service). Briefly, gas chromatography with flame ionization detection was used for the analysis of major volatile congeners, ultra‐high‐performance liquid chromatography for the analysis of maturation related congeners and ion exchange chromatography with pulsed amperometric detection for the analysis of carbohydrates. As the methods used by SWRI also have detection and quantification limits, not every sample of the 86 analyzed had a full set of congeners quantified. In addition, not all samples were analyzed for all congener classes (major volatile congeners, cask extractives, and carbohydrates). The number of analyzed samples for a particular congener is given in the first column of Table 1.

**TABLE 1:** Statistics of the analysis of 86 Scotch Whisky samples^a^, ^b^

	Analysed by SWRI	Profiled by ^1^H NMR	Below LOQ	Below LOD	Not seen^c^	>10% of nominal concentration^d^	Average relative difference from nominal concentration^e^, ^f^ ± SD (%)
Gallic acid	64	63	0	1	0	5	6.7 ± 4.8
Vanillin	68	32	0	27	9	8	9.9 ± 7.7
Vanillic acid	68	16	0	45	7	3	6.6 ± 6.2
Syringaldehyde	68	67	0	1	0	11	7.7 ± 6.9
Syringic acid	68	65	1	1	1	6	6.6 ± 6.5
Scopoletin	68	0	0	68	0	0	N/A
HMF	68	55	2	5	6	1	6.1 ± 4.4
Ethyl Acetate	85	76	4	5	0	1	5.2 ± 5.1
Methanol	71	71	0	0	0	4	6.1 ± 3.7
*n*‐Propanol	74	73	1	0	0	1	4.6 ± 2.9
iso‐Butanol	74	73	0	1	0	0	4.0 ± 3.4
Iso‐amyl acetate	74	73	0	1	0	59	38.3 ± 51.9
n‐Butanol	72	71	0	1	0	65	138.6 ± 504.1
2‐Methylbutanol	73	72	0	1	0	0	5.7 ± 3.4
3‐Methylbutanol	73	73	0	0	0	8	6.5 ± 3.8
Furfural	82	76	1	5	0	7	7.6 ± 5.9
Glucose	26	26	0	0	0	5	12.5 ± 15.8
Fructose	26	26	0	0	0	5	16.6 ± 23.8
Lactose	9	0	1	8	0	0	N/A
Sucrose	3	0	0	1	2	0	N/A
Maltose	3	0	0	3	0	0	N/A

a Not all 86 samples were profiled for all compounds. The number of congeners profiled is stated in the first column;

b Four congeners (scopoletin, lactose, sucrose, maltose) were below the LOD (or LOQ), hence results of only 17 congeners have average relative differences;

c The number of samples in which a compound should have been either detectable or quantifiable (i.e. the nominal concentration was above LOQ/ LOD by NMR) but was not present in the ^1^H NMR spectrum;

d The number of samples profiled with > 10% deviation from the nominal concentration.

e Average difference from nominal concentration expressed as % deviations include results that deviated > 10% for the nominal concentration.

f Average $\%Diff=\frac{1}{n}\left(\sum_{i=1}^{n}|\frac{NMR_{i}}{No\min al_{i}}\times 100-100|\right)\pm \sigma_{\%Diff}$.

### 2.2 Sample preparation and NMR experiments

The sample manipulation before NMR analysis was kept to a minimum to ensure that the whisky samples were not altered, and the sample preparation method developed by Kew et al^22^ was used. Briefly, 100 μL of a premade buffer was added to 500 μL of sample in a Norell (Norell, Inc, North Carolina, USA) S400 NMR tube. The buffer consists of sodium acetate‐d_3_/ acetic acid‐d_4_ made in D_2_O containing 6 mM of DSS‐d_6_ (3‐(Trimethylsilyl)‐1‐propanesulfonic acid‐d6 sodium salt) for use as an internal standard, yielding a final concentration of DSS of 1 mM and buffer of 25 mM at around pH 4.5 (Sodium acetate‐d_3_, D_2_O, and DSS‐d_6_ were sourced from Sigma‐Aldrich (Missouri, USA). Acetic acid‐d_4_ was sourced from Acros Organics (New Jersey, USA). NMR spectra were collected on a 600 MHz Bruker (Bruker Corporation, Massachusetts, USA) Avance III spectrometer equipped with a TCI cryogenically cooled probe, a 16‐position sample changer, and automatic tuning and matching. The suppression of water and ethanol, the two main components of whisky, was achieved according to Kew et al^22^ with a minimal suppression of signals near the ethanol or water resonances. This NMR method was specifically developed for the analysis of whisky and other spirits and consists of four experiments. In short, the first experiment locates the water signal, the second experiment determines the exact ^1^H chemical shift of the ^12^CH_2_ and ^12^CH_3_ signals of ethanol based on the analysis of their ^13^C satellites. The third experiment acquires the ^13^C spectrum and sets the parameters for the decoupling of the ^13^C satellites of ethanol. The fourth and final experiment is the acquisition of the ^1^H spectrum, using the parameters found by the previous three experiments, with water and ethanol signals suppressed. For water and ethanol presaturation, the power level was set to γB_1_/2π equal to 20 and 28 Hz, respectively. The receiver gain was kept at a constant value of 45.2, and spectra were obtained using four dummy scans and 32 scans. The FID was acquired with digitized sampling of 128k time domain points over 16 ppm, yielding the acquisition time of 6.82 s; a 4.5 s relaxation/presaturation delay was used. The FID was zero filled once and Fourier transformed using an exponential line broadening of 0.2 Hz. It took approximately 15 min to analyze one sample.

### 2.3 Data processing and targeted profiling

NMR spectra were processed using Mnova (Mestrelab, Santiago de Compostela, Spain). Each spectrum was manually phase corrected using a zero‐order phase correction only and baseline corrected via a Whittaker Smoother (filter 0.51 Hz, smooth factor 32768). The processed spectra (saved as .jdx files) were then imported into the Chenomx NMR Suite 8.4 (Chenmox, Alberta, Canada) processor software. An accurate concentration of DSS was assigned to its peak in each spectrum to allow absolute concentrations of congeners to be determined. The processed spectra were saved in the Chenomx proprietary software format and opened in Chenomx Profiler for targeted profiling.^23^ The software works by deconvoluting the whisky spectra using database spectra created from pure compounds acquired under identical (or similar) conditions as the targeted samples. The database spectra are signatures composed of peak clusters representing individual hydrogen environments of pure compounds. A Lorentzian peak shape was assumed for all peaks. Since the spectrum is complex, containing overlapping multiplets, a linear combination of all modeled analytes was used to produce a sum spectrum. Using DSS as the reference signal with a known concentration, the signals of interest were quantified.

## 3 RESULTS AND DISCUSSION

### 3.1 Custom compound selection and database creation

**FIGURE 1:**
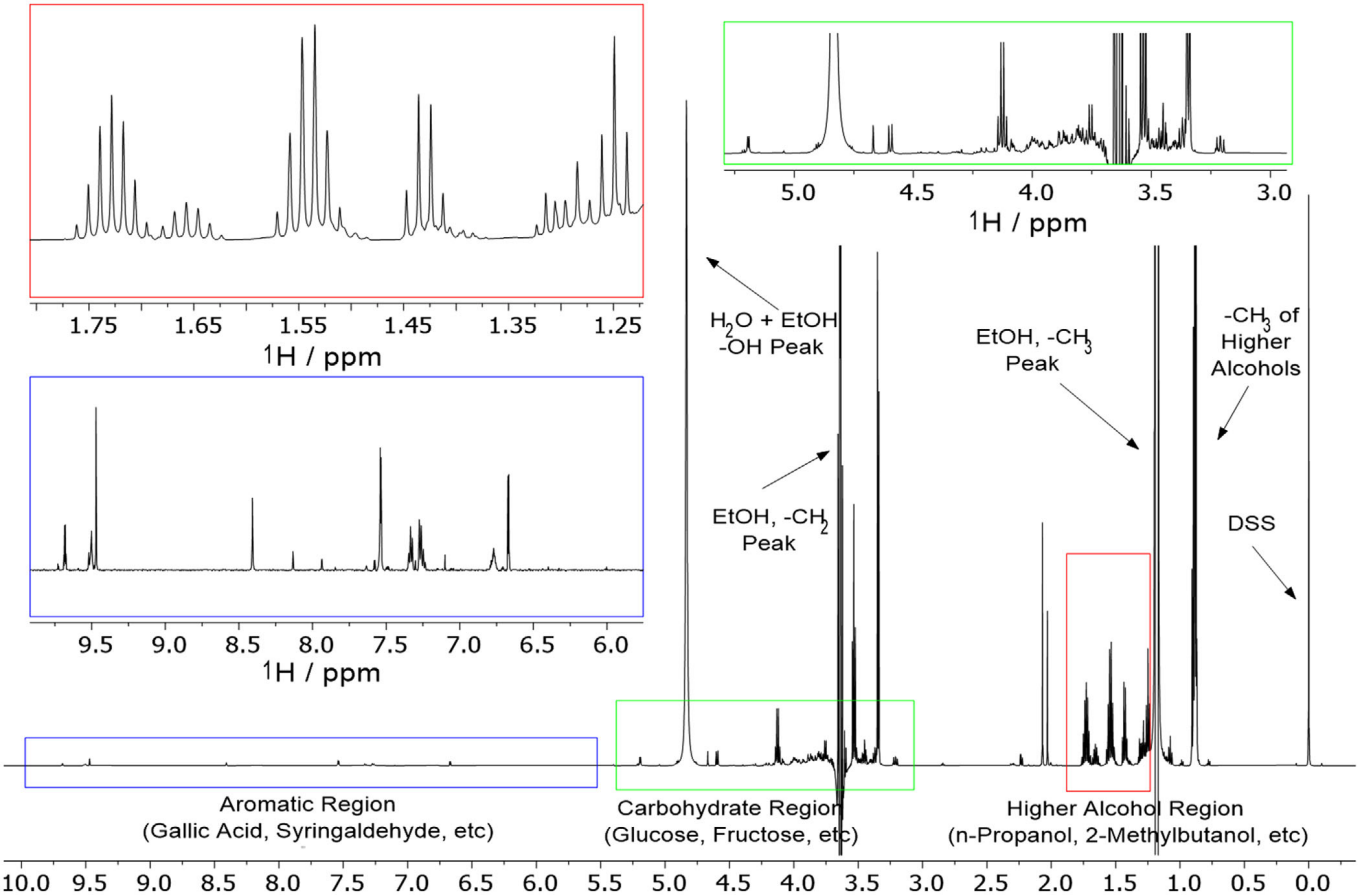
An example ^1^H NMR spectrum of a Scotch Whisky. Red box, higher alcohols; green box, carbohydrates; blue box, aromatics. Insets show vertical expansions of individual regions

Despite whisky containing thousands of compounds, as established by FT ICR MS,^24^, ^25^, ^26^, ^27^ ^1^H NMR spectra show signals of only a few dozen compounds. This is due to very different detection limits of the two techniques and can be viewed as a curse (loss of information) but also a blessing (possibility to quantify a limited number of the most concentrated compounds) by NMR. A typical ^1^H NMR spectrum of a Scotch Whisky is shown in Figure 1 and can be divided into three regions containing mostly signals of higher alcohols, carbohydrates, and aromatic compounds, respectively.

Twenty‐one compounds accessible by NMR were identified amongst routinely analyzed compounds in whisky and were the focus of this investigation. These included 14 compounds (gallic acid, syringaldehyde, 5‐HMF, ethyl acetate, methanol, *n*‐propanol, iso‐butanol, 2‐methylbutanol, 3‐methylbutanol, furfural and glucose, *n*‐butanol, syringic acid, and fructose) previously characterized by NMR by Kew et al.^18^ An additional seven compounds (vanillin, vanillic acid, scopoletin, iso‐amyl acetate, lactose, sucrose, and maltose) were added to this list and their resonances were assigned by analyzing spectra acquired in 40% EtOH solution (see Figure S1 and Table S1). In total, 21 compounds were thus investigated for quantification purposes. This is not a complete set of compounds identified by NMR in whisky samples by Kew et al.^18^ Some had to be excluded because of their minor presences or heavy overlap with signals of other (related) compounds (eg, phenolics) or because they are not routinely analyzed by the whisky industry. Further compounds, in principle amenable to NMR analysis such as (coniferaldehyde, sinapaldehyde, ethyl esters, etc), were not included for the same reasons.

**FIGURE 2:**
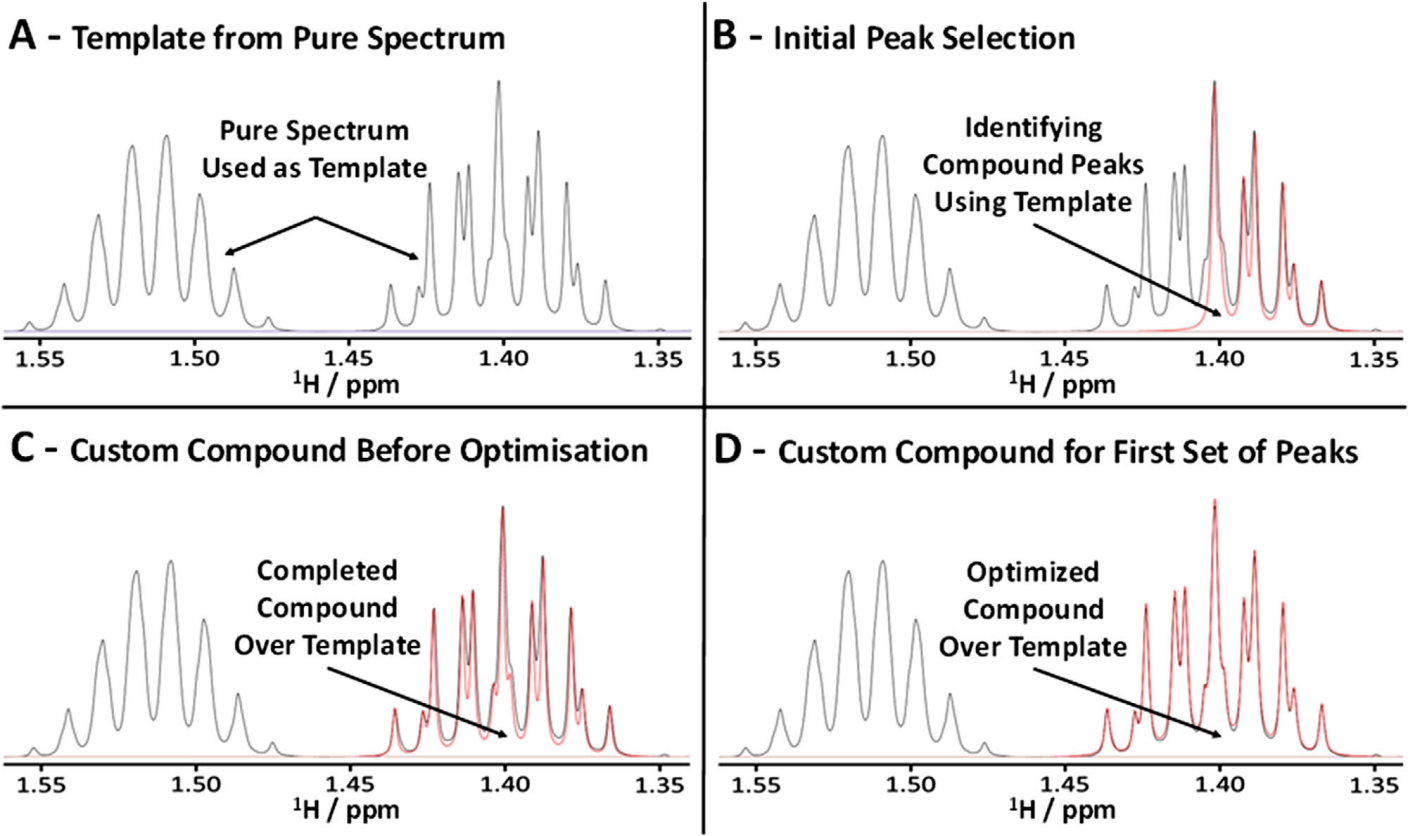
Example of a creation of a custom compound. A, Part of the experimental template for 2‐methyl‐1‐butanol; one of the H‐3 (1.40 ppm) and the H‐2 (1.51 ppm) signals are shown; B, Gradual building of the multiplet of H‐3 by identifying individual spectral lines; C, Completed multiplet of H‐3; D, Computer optimized multiplet of H‐3 matching the experimental template. Black, experimental template; red, custom compound signal

Chenomx NMR Suite 8.4 was used for the quantification of the analytes of interest. The standard spectral library of Chenomx is aimed at metabolomic analysis and therefore the majority of the compounds in the database are not relevant to this study. In addition, these are represented by spectra acquired in H_2_O and hence their chemical shifts differ, sometimes substantially, from those in alcohol/water mixtures. A custom compound building function of Chenomx was therefore used to build a library of 21 spectra acquired in 40% ABV solutions using the custom solvent suppression method.^22^ It was possible to accurately profile samples >40% ABV using the same custom compounds spectra; separate sets of samples at differing %ABV were not needed. Inclusion of a custom compound starts by the acquisition and processing of its template spectrum as described in Section 2, and attributing its concentration based on the integral of the DSS signal. The process of the construction of the custom compound is illustrated in Figure 2 for H‐3 of 2‐methyl‐1‐butanol (δ = 1.40 ppm). At this point, signals that are in poorly resolved regions of whisky spectra and signals near the ethanol and water signals can be omitted from the custom compound. This typically concerns analytes resonating in regions 1.05‐1.30 ppm (EtOH, CH_3_ peak), 3.45‐3.85 ppm (EtOH, CH_2_ peak), and 4.6‐5.2 ppm (EtOH and H_2_O, OH peak). In our analysis, we also omitted the methyl signals of congeners because they all overlap in a very narrow window of 0.8‐0.95 ppm.

### 3.2 Analysis of model mixtures

To ensure that the custom compounds in the database were accurately defined and to establish if they profile compounds in mixtures accurately and robustly, they were first tested in a controlled environment. Twenty‐one different model mixtures were prepared containing between 2 and 13 compounds using a range of concentrations reflecting their relative presence in whisky (Table S2). These yielded some spectra with no overlapping peaks and others with peaks that overlapped with varying degrees of severity. For overlapping multiplets, it was investigated whether the compounds had to be profiled in any particular order. It was concluded that for the bulk of the compounds, the order of profiling did not matter, except for carbohydrates. It was found that the most consistent and accurate results were obtained when these compounds were profiled in the following order: sucrose, maltose, lactose, fructose, and glucose; the most important in this process was including glucose as the last compound to be profiled. The results of this analysis are presented in Table S3. With the exception of iso‐amyl acetate and *n*‐butanol, all compounds in all mixtures were profiled to ≤10% from their nominal values. The iso‐amyl acetate and *n*‐butanol only showed >10% deviations in the model mixtures with severe overlap of intense signals from other compounds. In fact, most of the compounds showed an agreement to around 5% with the exception of glucose and methanol. The poorer agreement observed for glucose is likely due to its protons resonating close to the suppressed OH and CH_2_ water/ethanol signal. Glucose signals could be affected by partial saturation or minor baseline distortions caused by “solvent” suppression and/or overlap with signals of other carbohydrates. Deviations in methanol concentration could be due to possible fluctuation of methanol as a contaminant in ethanol used (discussed later), especially since the achieved agreement for Scotch Whisky samples was better (compare Table S3 and Table 1).

### 3.3 Limit of quantification and limit of detection

Determining the limit of quantification (LOQ) and limit of detection (LOD) is part of the profiling process ensuring accuracy at low concentrations. These parameters were determined by making a series of dilutions from a concentrated solution of known strength and acquiring data until the resulting spectrum fell out with set limits. The compound was considered no longer quantifiable once the predicted concentration had a percentage deviation from the nominal value (Equation 1) greater than ±10%.

(1) $$\%\;\mathrm{Devation}=\left(\frac{(\mathrm{Determined}\;\mathrm{value}\;\times \;100)}{\mathrm{True}\;\mathrm{Value}}\right)-100\;$$

The compound was considered detectable only when all the peaks of the compound were present at a signal‐to‐noise ratio (SNR) > 4. This exercise was conducted for all 21 compounds with the resulting LOQ and LOD values shown in Table 2.

**TABLE 2:** Summary of the LOQ and LOD values for the investigated compounds at 600 MHz Bruker Avance III spectrometer equipped with a TCI cryogenically cooled probe

	LOQ Value (μM)	LOD Value (μM)	LOQ Value (mg/L)	LOD Value (mg/L)
Gallic Acid	1.22	0.87	0.21	0.15
Vanillin	5.22	4.70	0.79	0.71
Vanillic Acid	5.28	4.32	0.89	0.73
Syringaldehyde	1.53	0.85	0.28	0.15
Syringic Acid	0.87	0.73	0.17	0.14
Scopoletin	4.56	4.18	0.88	0.80
HMF	4.42	3.69	0.56	0.47
Ethyl Acetate	12.8	3.68	1.12	0.32
Methanol	27.3	13.4	0.87	0.52
*n*‐Propanol	22.9	14.6	1.38	0.88
Iso‐Butanol	15.8	11.8	1.12	0.87
iso‐Amyl Acetate	19.1	15.3	2.48	1.99
*n*‐Butanol	15.1	11.8	1.12	0.87
2‐Methylbutanol	34.1	31.2	3.00	2.75
3‐Methylbutanol	19.9	12.8	1.76	1.13
Furfural	10.5	7.85	1.01	0.75
Glucose	31.1	28.9	5.61	5.21
Fructose	101	20.2	18.2	3.64
Lactose	30.6	16.5	10.5	5.64
Sucrose	16.4	11.7	5.61	4.01
Maltose	21.1	11.8	7.24	4.02

It can be seen that the LOQ/ LOD for the maturation related congeners (gallic acid, vanillin, etc) are lower (micromolar concentration) than those of the major volatile congeners (*n*‐propanol, iso‐butanol, etc) (10‐20 μM) and carbohydrates (glucose, fructose, etc) (10‐100 μM). This is because the spectra of major volatile congeners and carbohydrates are composed of multiplets, while the maturation related congeners containing more isolated protons show simpler multiplets or singlets resulting in higher SNR, making it easier to detect and quantify them.

The LOQ (27.3 μM) and LOD (16 μM) values of methanol are relatively high compared to the other alcohols (whose spectra are made up of multiplets), and much higher than those of maturation related congeners whose signals are mainly doublets or singlets. This is due to an unavoidable methanol contamination of the EtOH solvent used to prepare both the standard solutions and model mixture samples, whose concentration (16 μM) was determined using reference deconvolution in MestRenova. This value was subtracted when profiling whisky samples. Nevertheless, the presence of this contamination meant that the LOD had to be set at 16 μM as there was no reliable way to determine concentrations below this value.

### 3.4 Analysis of whisky spectra

**FIGURE 3:**
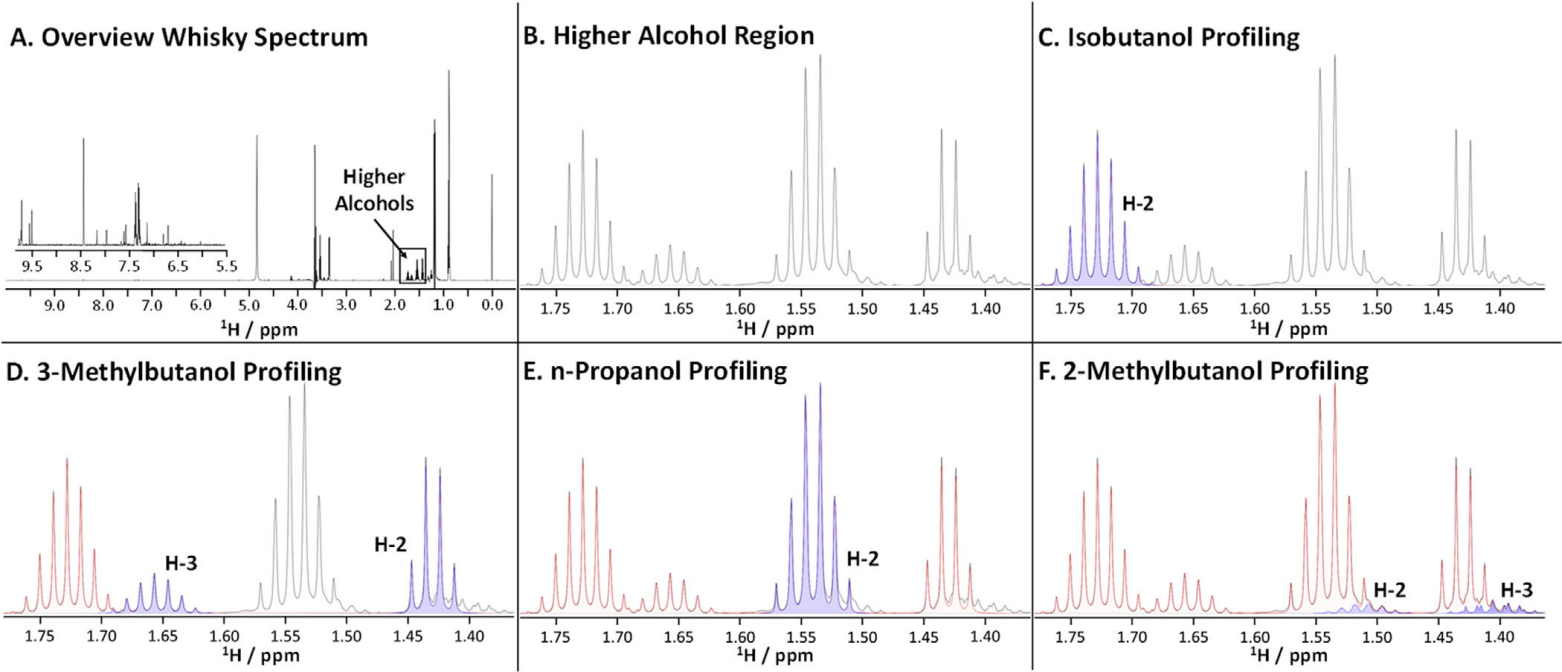
Example of the workflow for profiling a Scotch Whisky sample using Chenomx NMR suite. A, Full ^1^H NMR spectrum; B, higher alcohols region; Profiling of (C) H‐2 multiplet of isobutanol; D, H‐2 and H‐3 multiplets of 3‐methylbutanol; E, H‐2 multiplet of n‐propanol; F, H‐2 and H‐3 multiplets of 2‐methylbutanol. Blue peaks indicate the compound being profiled; the red line is the summation line

Once the custom database containing compounds to be profiled had been created, whisky samples were prepared, their spectra acquired, processed, and imported into Chenomx profiler software. Figure 3 shows an example workflow of the Chenomx profiling process for the determination of the absolute concentration of whisky congeners.

Both automatic and manual deconvolutions of the spectra are possible. In this study, the spectra were initially profiled automatically before manual fine‐tuning ensured an optimal fit of resonances. In case of severe overlap, and particularly when large differences in the signal intensity were observed, a “handle signal” was identified for each compound (Table S4). Such a signal was well resolved and sufficiently distant from the solvent suppression sites, and hence not affected by partial saturation or occasional baseline distortions. Since the intensity of all signals of a custom compound increase at the same rate, it is sufficient to focus on one signal and put less weight on the other, possibly compromised signals. Using this approach, increased accuracy of the profiling compared to automated fitting was achieved.

### 3.5 Profiling a set of whisky samples

To evaluate the profiling performance of the developed custom ^1^H NMR database, 86 Scotch Whisky samples were profiled. These had been also analyzed by SWRI using UKAS accredited non‐NMR based methods of analysis. The samples were prepared, acquired, and processed in the same way as those of the pure compounds and the model mixtures. Profiling of individual regions of a ^1^H NMR whisky spectrum in Chenomx is illustrated in Figure S2, and the results are summarized in Table 1 containing a comparison of the ^1^H NMR and the SWRI analysis of samples of 21 congeners.

Excluding six compounds discussed below, it was possible to analyze the remaining 15 congeners in 81‐100% of samples by both methods. Among the six compounds, vanillin and vanillic acid were profiled by NMR only in 47% and 24% of samples, respectively, while scopoletin, lactose, sucrose, and maltose could not be profiled in any sample. This is because they were present below the LOD of NMR. The three carbohydrates mentioned are routinely analyzed by the SWRI, but were detected only in a very small sample set (3‐9 out of 26), reflecting their limited concentrations in whisky as measured using the ion exchange method. These carbohydrates were accurately quantified in model mixtures, therefore at higher concentrations scopoletin, lactose, sucrose, and maltose can in principle be accurately quantified in whisky samples.

Table 1 also presents the number of samples where NMR values for individual compound deviated >10% from their nominal concentrations. The percentage of such samples varied between 0 (iso‐butanol or 2‐methylbutanol) and 25% (vanillin), with the exception of iso‐amyl acetate and *n*‐butanol, which showed the poorest performance among the 21 investigated congeners. This is because signals of these low concentration compounds overlap with signals of more concentrated compounds, such as higher alcohols (2‐methylbutanol, 3‐methylbutanol, *n*‐propanol) or ethyl acetate. A similar observation was also made in experiments on model mixtures or attempts to quantify ethyl carbamate in a previous study.^21^ For 16‐25% of the NMR analyzable samples, vanillin, vanillic acid, and syringaldehyde also returned concentrations >10% from the nominal values. For vanillin and vanillic acid, this is because in most samples their concentration was close to LOD. As for syringaldehyde, the deviations are likely due to the proximity of its main signal to two multiplets of 2‐phenylethanol (not analyzed in this study). The two carbohydrates, glucose and fructose, which could be quantified, did show a >10% deviation from their nominal values in 19% of analyzed samples for the reasons discussed under analysis of model mixtures. For whisky samples, the situation was further aggravated by overlap with additional signals from non‐carbohydrate compounds.

The relative differences between the nominal concentrations of whisky congeners and NMR determined values indicate a good overall agreement for the majority of the 21 compounds (Table 1). Excluding iso‐amyl acetate, *n*‐butanol, glucose, and fructose, the average relative difference of 6.4% was obtained with an average standard deviation of 5.0% showing the accuracy and consistency of the NMR method presented here. Iso‐amyl acetate and *n*‐butanol signals were dwarfed by more intense, overlapping signals of other compounds. Analysis of carbohydrates was complicated by mutual signal overlap and while performing well in model mixtures, the results were less satisfactory for whisky samples. Some congeners such as vanillin, vanillic acid, and scopoletin, when present in whisky samples at concentrations below the LOD and LOQ amounts, could not be quantified.

## 4 CONCLUSIONS

This work has demonstrated that, combined with high quality water and ethanol signals suppression, cryoprobe ^1^H NMR at 600 MHz has a great potential for targeted quantification of Scotch Whisky congeners. The main issue that complicates quantitative analysis of whisky compounds – an overlap of NMR signals – was addressed by using Chenomx NMR software, which provided reliable deconvolution of resolved, but overlapping signals. LOQ and LOD were established for all 21 studied congeners ranging from milligrams to sub‐milligrams of compounds per liter. High‐field NMR represents a one‐stop method for quantification of a number of frequently analyzed whisky congeners. In situations where high volume of samples need to be analyzed, minimum sample preparation and high throughput (approximately 15 min/sample), can offset high acquisition and maintenance costs of NMR instrumentation.

## CONFLICT OF INTEREST

MS was funded, in part, by SWRI; IG is employed by SWRI. SWRI is the Scotch Whisky industry's Research & Technology Organisation; it is funded by its membership of Scotch Whisky production companies. SWRI's remit is to ensure sustainability of the industry and its supply chain, improve process efficiency and help protect the category. It does this by carrying out a comprehensive program of precompetitive and applied research.

## Supporting information

Supporting Information

## Data Availability

NMR data discussed in this paper are available from: http://hdl.handle.net/10283/3703
