# Supplementary material for: Quantification of whisky congeners by 1H NMR spectroscopy
Source: Anal Sci Adv. 2020 Jul 27;1(2):132–40. doi: 10.1002/ansa.202000063 (PMC10989066; doi:10.1002/ansa.202000063)
Supplement: Supplementary file 1 — Supporting Information [file ANSA-1-132-s001.pdf]

Supporting Information

Quantification of Whisky Congeners by ^{1}H NMR Spectroscopy

Marc Stockwell,[a] Ian Goodall,[b] Dušan Uhrín[a,*]

[a]EaStCHEM School of Chemistry, Joseph Black Building, University of Edinburgh, David Brewster Rd, Edinburgh, UK, EH9 3FJ.

[b]The Scotch Whisky Research Institute, The Robertson Trust Building, Research Avenue North, Riccarton, Edinburgh, UK, EH14 4AP.

Table of content:

Figure S1: ^{1}H NMR spectra of seven whisky congeners……………………………………………………………………………...............2

Table S1: ^{1}H NMR parameters of seven whisky congeners………………………………………………………………………………. 3

Table S2: Composition of model mixtures (mM) used for testing of the quantification protocol………………………………………………………4

Table S3: Comparison of the nominal and NMR derived concentrations of whisky congeners in 21 model mixtures……………………………5

Table S4: Handle peaks for individual compounds……………………………………………………………………….……………...6

Figure S2: Profiling of the ^{1}H NMR spectra of Scotch Whisky..……………………………………………………………….…….7

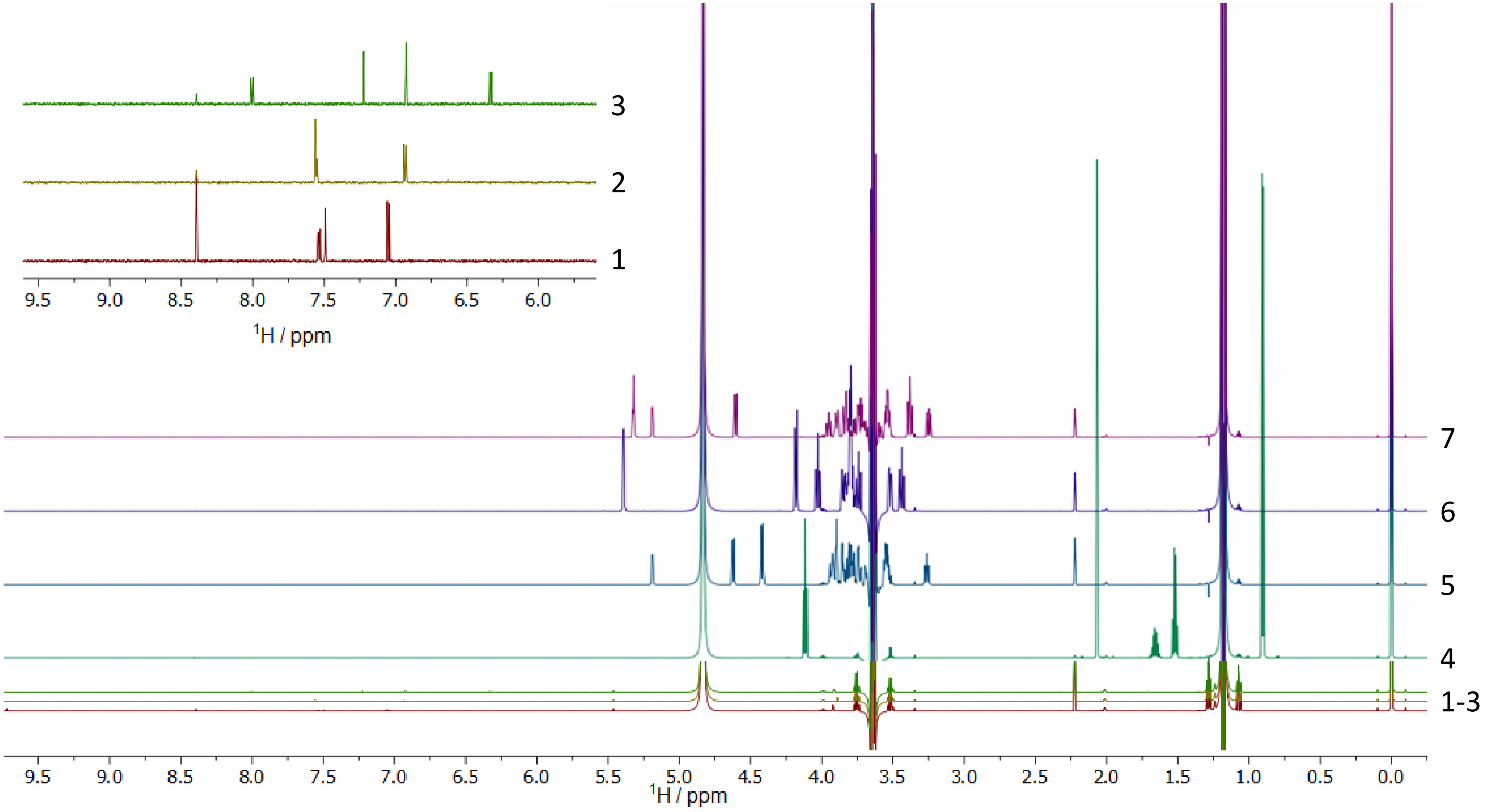

Figure S1. ^{1}H NMR spectra of seven compounds previously not characterised by Kew *et al* (2019). 1 - vanillin; 2 - vanillic acid; 3 – scopoletin; 4 – iso-amyl acetate; 5– lactose; 6 – sucrose; 7 – maltose. The inset contains aromatic regions of the spectra for compounds 1-3.

Table S1. ^{1}H NMR parameters of additional compounds profiled in Scotch Whisky by NMR, which were not characterised in Kew *et al* (2019), including ^{1}H chemical shifts, *J* coupling constants, multiplicity (m), group, and number of protons. For lactose, sucrose and maltose only the key signals used in the profiling are listed.

Compound	δ(^{1}H)/ ppm	J / Hz	Multiplicity	Group	Number of protons	Compound	[1]H / ppm	Multiplicity
Vanillin 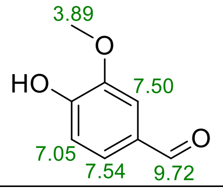	3.89	-	s	CH_3	3	Lactose	3.26	t
	7.05	8.01	d	CH	1		3.90	d
	7.50	1.85	d	CH	1		4.42	d
	7.54	8.20, 1.88	dd	CH	1		4.62	d
	9.72	-	s	CH	1		5.19	d
Vanillic Acid 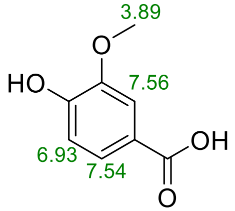	3.89	-	s	CH_3	3	Sucrose	3.44	t
	6.93	8.20	d	CH	1		3.52	dd
	7.54	8.07, 2.0	dd	CH	1		4.30	t
	7.56	2.02	d	CH	1		4.18	d
							5.39	d
Scopoletin 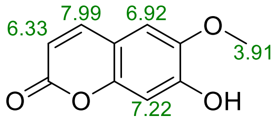	3.91	-	s	CH_3	3	Maltose	3.95	t
	6.33	9.41	d	CH	1		4.60	d
	6.92	-	s	CH	1		5.19	d
	7.22	-	s	CH	1		5.32	t
	7.99	9.48	d	CH	1			
Iso-Amyl Acetate 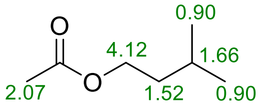	0.90	6.61	d	CH_3	6			
	1.52	6.82	q	CH_2	2			
	1.66	6.79	m	CH	2			
	2.07	-	s	CH_3	3			
	4.12	6.80	t	CH_2	2			

Table S2. Composition of model mixtures used for testing of the quantification protocol. Concentrations are given in mM.

Mixture number (overlap)*	1(1)	2(3)	3(5)	4(2)	5(2)	6(1)	7(1)	8(5)	9(1)	10(3)	11(2)	12(1)	13(2)	14(2)	15(5)	16(1)	17(4)	18(4)
Gallic Acid						0.0105			0.0105			0.0070			0.0035	0.0035		
Vanillin																0.0042		
Vanillic Acid																0.0038		
Syringaldehyde							0.0097		0.0097		0.0065	0.0065			0.0032	0.0034		
Syringic Acid							0.0061		0.0061		0.0061	0.0061			0.0031	0.0029		
Scopoletin																0.0030		
HMF							0.0184				0.0138	0.0138			0.0055	0.0049		
Ethyl Acetate								1.14			1.14				0.455		1.14	1.1
Methanol						0.94									0.312			
n-Propanol	1.67		1.67					1.66		1.66					0.664			
iso-Butanol	1.35		1.35					1.35							0.539			
iso-Amyl Acetate								0.0767		0.0767	0.0762				0.0307		0.0572	0.193
n-Butanol		1.35	1.35					0.0675		0.0675					0.0227			
2-Methylbutanol		1.13	1.13	1.13	0.57			0.566					4.53	0.564	0.0227			
3-Methylbutanol	1.13		1.13	0.57	1.13			1.13					0.565	4.52	0.452			
Furfural											0.104	0.104			0.0417			

Mixture Overlap	19(5)	20(5)	21(4)
Glucose	0.455	0.455	
Fructose	0.423	0.423	
Lactose	0.415	0.0415	0.0094
Sucrose	0.457	0.0457	0.0094
Maltose	0.426	0.0426	0.0094

*Numbers 1-5 given in parenthesis indicate severity of the overlap. 1 - no overlap, 5 - severe overlap.

Table S3. Comparison of the nominal and NMR derived concentrations of whisky congeners in 21 model mixtures[a]

Compound	*Number of compounds in the mixture*	*Profiled successfully by ^1H NMR*	*No of >10% deviations from the nominal value*	*Average relative difference from the nominal concentration[b] ± stdev (%)*
Gallic acid	5	5	0	4.5 ± 3.6
Vanillin	1	1	0	5.1 ± 0.0
Vanillic acid	1	1	0	6.3 ± 0.0
Syringaldehyde	6	6	0	4.6 ± 4.2
Syringic acid	6	6	0	5.5 ± 3.7
Scopoletin	1	1	0	2.7 ± 0.0
HMF	5	5	0	3.4 ± 3.5
Ethyl acetate	5	5	0	4.9 ± 2.9
Methanol	2	2	0	10.2 ± 0.5
n-Propanol	5	5	0	5.2 ± 2.9
iso-Butanol	4	4	0	2.2 ± 2.2
iso-Amyl acetate	6	6	4	23.5 ± 19.8
n-Butanol	5	5	2	16.2 ± 9.5
2-Methylbutanol	7	7	0	4.5 ± 2.8
3-Methylbutanol	8	8	0	5.7 ± 3.6
Furfural	4	4	0	3.7 ± 2.9
Glucose	2	2	0	10.6 ± 0.2
Fructose	2	2	0	1.5 ± 1.2
Lactose	3	3	0	5.7 ± 1.4
Sucrose	3	3	0	7.9 ± 1.9
Maltose	3	3	0	6.2 ± 0.2

[a]Average difference from nominal concentration expressed in % deviations include results that deviated >10% for the nominal concentration.

[b]Average % Diff $= \frac{1}{n}\left(\sum_{i=1}^{n}\left|\frac{NMR_i}{Nominal_i} \times 100 - 100\right|\right) \pm \sigma_{\%Diff}$

Table S4. Handle peaks for individual compounds.

Compound	*δ(^{1}H)/ ppm*	*Hydrogen type*	*Multiplicity*
Gallic Acid	7.10	CH	singlet
Vanillin	7.50	CH	doublet
Vanillic Acid	6.93	CH	doublet
Syringaldehyde	7.30	CH	singlet
Syringic Acid	7.32	CH	singlet
Scopoletin	6.33	CH	doublet
Hydroxymethylfurfural	7.54	CH	doublet
Ethyl Acetate	4.13	CH_2	quartet
Methanol	3.35	CH_3	singlet
n-Propanol	1.54	CH_2	sextet
iso-Butanol	3.35	CH_2	doublet
iso-Amyl Acetate	4.12	CH_2	triplet
n-Butanol	1.52	CH_2	pentet
2-Methylbutanol	3.36	CH_2	higher order multiplet
3-Methylbutanol	1.66	CH	septet
Furfural	7.93	CH	doublet of triplets
Glucose	5.19	CH	doublet
Fructose	4.08	CHs	overlapping multiplets
Lactose	4.42	CH	doublet
Sucrose	5.39	CH	doublet
Maltose	5.32	CH	triplet

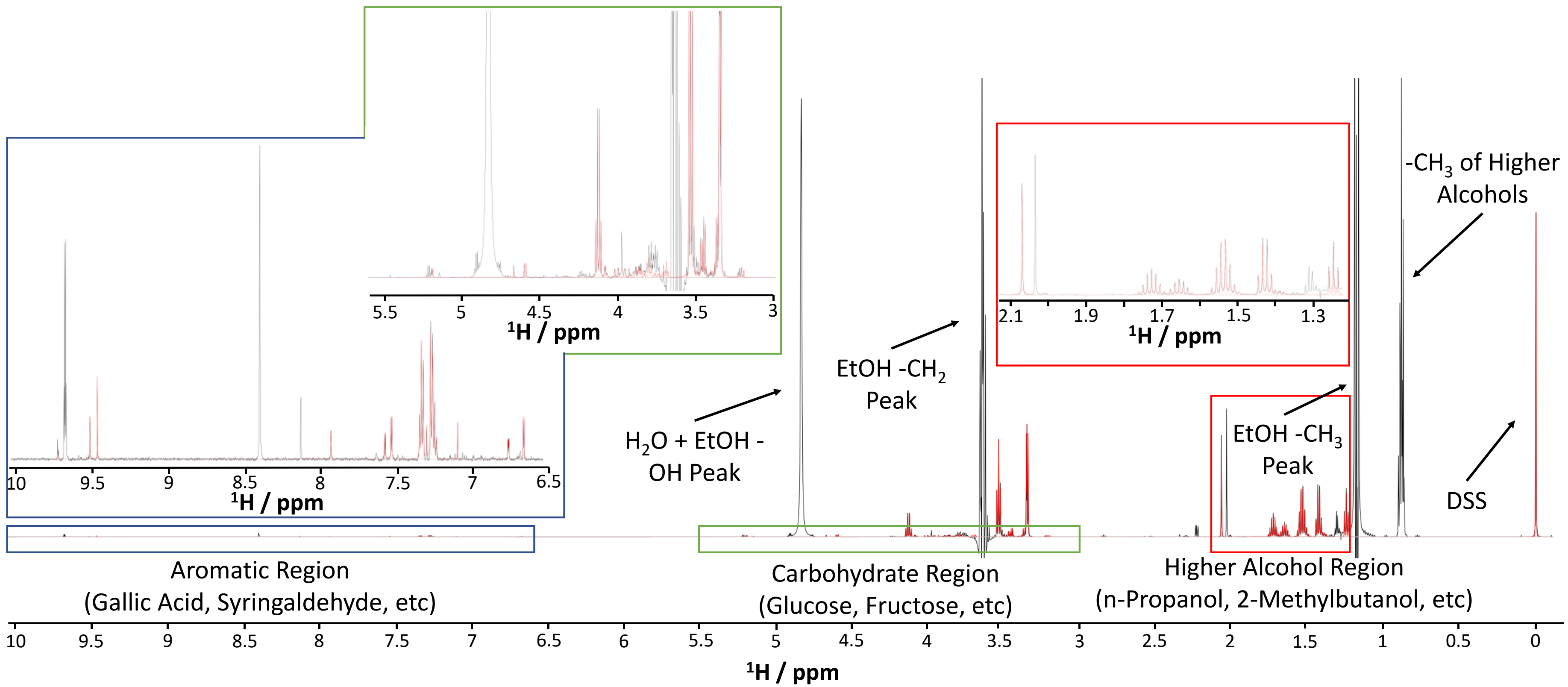

Figure S2. Profiling of the three regions of ^{1}H NMR spectra of Scotch Whisky. Red box – higher alcohols, green box – carbohydrates, blue box – aromatics.
